# Exploring challenges to medication adherence aacmong young and middle-aged adults with coronary heart disease in China: a qualitative study

**DOI:** 10.3389/fcvm.2025.1664013

**Published:** 2025-10-30

**Authors:** Jianli Guo, Jian Ma, Mengqi Xu, Xiaoli Huang, Yuan Ding, Lingyan Zhu, Wengui Zheng

**Affiliations:** ^1^ Department of Nursing, Shanghai Sixth People's Hospital Affiliated to Shanghai Jiao Tong University School of Medicine, Shanghai, China; ^2^ The Nethersole school of Nursing, Faculty of Medicine, The Chinese University of Hong Kong, Hong Kong SAR, China

**Keywords:** young and middle-aged, coronary heart disease, medication adherence, secondary prevention, qualitative study

## Abstract

**Background:**

Although coronary heart disease (CHD) is becoming increasingly common among young and middle-aged adults, their outcomes usually remain suboptimal. One major contributing factor is poor medication adherence. This study aims to explore the challenges they face in maintaining adherence to prescribed medications.

**Methods:**

This was a qualitative study. 27 young and middle-aged adults with CHD (20 men and 7 women) were recruited from a tertiary general hospital in Shanghai between March and May of 2025. Data were collected through semi-structured interviews. An inductive thematic analysis approach was used for data analysis.

**Results:**

Two themes and four sub-themes were identified in total: (1) Struggling with Illness Identity and Long-Term Medication: Viewing CHD as an acute condition; Suffering from social isolation due to CHD. (2) Taking Medication as an Intruder in Life: Disrupting daily routines due to medications; Experiencing conflicts between medication taking and social responsibility.

**Conclusion:**

Young and middle-aged adults with CHD may have difficulty maintaining medication adherence due to distorted illness perceptions, emotional resistance to accepting a chronic condition, and the marginalization of medication-taking within their busy daily routines. These findings highlight the need to explore psychological approaches, such as Acceptance and Commitment Therapy (ACT), as potentially relevant strategies to support illness acceptance and psychological adjustment in this demographic. In addition, nurse-led initiatives that facilitate the integration of medication-taking into daily life warrant further investigation. Future mixed-methods and interventional studies are needed to test the feasibility and effectiveness of these approaches.

## Introduction

Coronary heart disease (CHD) caused approximately nine million deaths globally in 2019, accounting for 16% of all deaths worldwide (1–3). By 2050, CHD is projected to become the most prevalent cardiovascular disease (4). CHD has increasingly affected young and middle-aged adults in recent years (5–7). In the United States, 15.7% of adults under the age of 60 were living with CHD (8). In China, young and middle-aged adults account for between 27.1% and 46.7% of all acute myocardial infarction (AMI) cases (9–11), and their hospitalization rates have steadily increased (12). Importantly, China ranks among the countries with the highest age-specific burden of CHD-related disability-adjusted life years (DALYs) in adults aged 25 to 49 (13).

CHD in young and middle-aged adults often presents with few or no early symptoms, which makes it less likely to be recognized and managed in time. When risk factors are poorly controlled, the disease may progress rapidly, thereby increasing the risk of adverse outcomes (14–16). Guidelines from AHA and ESC strongly recommend the long-term combination therapy with four drugs to control risk factors associated with the progression of CHD: antiplatelet agents, renin–angiotensin–aldosterone system inhibitors, beta-blockers, and hypolipidemic agents (17, 18). Medication adherence is defined as the extent to which a patient's medication-taking behavior aligns with the regimen prescribed by their healthcare provider (19). Poor adherence is currently regarded as a major factor contributing to the discrepancy between the recommended and actual use of secondary prevention therapies in CHD (20). Medication nonadherence diminishes the effectiveness of drug therapy, leading to adverse health outcomes and increased healthcare costs (21, 22). Improving adherence has long been a priority for healthcare systems. However, young and middle-aged adults with CHD consistently demonstrate lower medication adherence compared to older adults (23–25). This highlights the urgent need to understand their specific challenges when it comes to maintaining medication adherence (26).

According to the World Health Organization (WHO), cardiovascular diseases account for 38% of all premature deaths globally (27). Although CHD affects more young and middle-aged adults, existing research on medication adherence has largely focused on older adults. Only a limited number of studies about young and middle-aged adults have examined factors such as self-efficacy, social support and side effects (28–31). However, these studies tend to remain at the level of variable-based analysis and fall short of offering deeper insight into the underlying reasons for changes in adherence behavior.

Compared to older adults, young and middle-aged adults often face heavier social and psychological burdens. Many of them are managing demanding jobs, raising children, and supporting aging parents at the same time (32, 33). Between 20% and 75.5% of them experience ongoing psychosocial challenges (34), including chronic stress, maladaptive coping strategies, and low self-esteem (35), which may reduce the mental and emotional capacity required to consistently manage medication-taking behaviors (36, 37). Such findings imply the existence of other overlooked factors that could substantially impact on medication adherence among young and middle-aged adults with CHD. These factors are difficult to capture through surveys or other quantitative methods.

Therefore, this study seeks to understand the everyday challenges that young and middle-aged adults with CHD face in adhering to medications, focusing on how they interpret these experiences within the context of their daily lives.

## Materials and methods

### Design

This study employed a qualitative approach (38). Semi-structured interviews were conducted with participants to explore the challenges of their medication adherence. The findings are reported in accordance with the Consolidated Criteria for Reporting Qualitative Research (COREQ) (39) (Supplementary Appendix S1).

### Setting and participants

Participants were recruited through purposive sampling based on maximum variation, targeting diversity in sex, age, myocardial infarction history, CHD history, treatment class (surgical or medical), marital status, education, occupation, household registration, and type of health insurance. The study was conducted from March to May of 2025 in the cardiology wards and outpatient clinics of a tertiary hospital in Shanghai, China. The inclusion criteria for participants included (1) be aged 18 to 60 years (40–42) and (2) be diagnosed with CHD. As the prevalence of CHD among individuals under 40 is only 1.7% (8), no participants aged 18–34 were enrolled during the recruitment. Patients with unstable clinical conditions or those enrolled in other interventional studies were excluded. Eligible participants were identified through the hospital's electronic medical records. The researcher, in her role as a nursing student, then approached them during routine ward and clinic care to explain the study. Of the 35 approached, 27 met the eligibility criteria consented and completed interviews. All participants were informed of the study's objectives, confidentiality measures and provided written informed consent.

### Data collection

Guo, a master's nursing student trained in qualitative research, developed the initial interview guide based on a review of relevant literature (19, 28–31, 43, 44) (see Supplementary Appendix S2) and conducted two pilot interviews to refine it. As the guide was still being tested at this stage, these pilot sessions were generally shorter (25–50 min), and data from them were excluded from the formal analysis. Thereafter, Guo carried out all formal interviews independently in a private ward. With participants' consent, Guo recorded the interviews and took field notes, paying close attention to nonverbal cues such as body language, facial expressions, and tone of voice. The formal interviews varied in length from 30 to 90 min, as participants' experiences and narratives differed in complexity and depth. After 24 interviews, only additional instances of existing codes were identified, and no new codes or themes emerged, indicating that data collection had reached saturation (45). Three further interviews were conducted to confirm that no new themes would arise. Data collection was therefore concluded after the 27th interview. Theme stability was subsequently verified through team discussions.

### Data analysis

We imported the transcribed textual material into MAXQDA 2020 and analyzed using an inductive thematic analysis approach (46). Guo conducted the initial analysis independently, following the thematic analysis steps: (1) The recordings were listened to repeatedly and the transcripts were read thoroughly, with initial thoughts noted; (2) Initial codes were generated by examining the transcripts line by line, openly coding meaningful words and phrases, and organizing data pertinent to each code; (3) The codes were subsequently merged into potential categories, collecting all data related to these themes; (4) Themes were reviewed, with merging, modification, or deletion based on criteria of internal homogeneity and external heterogeneity; (5) Themes were defined and named; (6) Qualitative results reports were written and analytical findings presented in a structured format. After Guo developed the initial themes, Xu independently reviewed all codes, subthemes, and themes against the transcribed textual material. Any discrepancies were discussed within the research team until consensus was reached. A coherent set of clearly defined themes was developed to capture participants’ experiences and perspectives through 3 iterative rounds. Throughout the analysis, a versioned codebook, analytic memos, and meeting minutes were maintained to ensure transparency and dependability of the analytic process.

### Ethical considerations

This study was approved by the Ethics Committee of Shanghai Jiao Tong University Affiliated Six People's Hospital on February 22, 2025, under approval number 2025-KY-041(K). All participants were informed about the study's purpose and procedures, assured of confidentiality, and provided written informed consent before the interviews. They were free to decline any question or withdraw from the study at any time without consequence. All procedures involving human participants followed the principles of the Declaration of Helsinki. As this was a qualitative study, trial registration was not applicable.

### Rigor

This study adhered strictly to the principles of reliability, credibility, and transferability in qualitative research (47). Prior to data collection, the interviewer received systematic training in qualitative methodologies and avoided leading questions. Before analysis, transcripts were returned to participants for proofreading; one participant provided feedback, and their data were revised accordingly. During data analysis, Guo independently conducted the coding, and a doctoral nursing student Xu subsequently reviewed all codes, subthemes, and themes. Zhu, a graduate advisor with extensive qualitative research experience, provided methodological guidance throughout the research process to ensure the accuracy of theme development and the overall credibility of the findings. The coding framework underwent 3 iterations, with the codes and categories being refined at each stage. Discrepant or negative cases were discussed in team meetings, during which Guo and Xu revisited the recordings and explored alternative interpretations. For instance, in the third iteration, Xu split the code “Refilling meds is burdensome” into “Time-consuming process of medication refills” and “Financial burden of medication refills”, reflecting two different aspects of the issue. Guo agreed with this revision. When consensus could not be reached, Zhu facilitated resolution. Reflexivity during data collection and analysis was documented to ensure transparency and rigor (Supplementary Appendix S5). For translation, Guo and Xu independently translated the selected quotations into English. Guo had passed the CET-6 exam, while Xu had passed the IELTS exam and had studied at an English-medium university. Both had sufficient translation competence. The translation emphasized the accuracy of medical and nursing terminology as well as cultural appropriateness. Zhu, a nursing scholar with an IELTS certificate, reviewed the drafts, resolved semantic differences, and produced a harmonized version. Yi Zhou, a native English speaker who was not involved in the study design or data collection, back-translated the quotations into Chinese. The back-translated versions were compared with the original transcripts to confirm semantic equivalence, any discrepancies were resolved through one team discussion. The final English quotations are presented in the Supplementary Appendix S3 (see also Supplementary Appendices S5, S6 for the reflexive statement and the translators' qualifications). Additionally, detailed descriptions of the study design, participant criteria, data collection were provided. This process strengthened all aspects of the reliability, credibility, and transferability of the findings.

## Results

A total of 27 young and middle-aged adults with CHD participated in this study. Among them, there were 20 males and 7 females, aged between 35 and 58 years. Adults aged 18–34 were eligible, but none were enrolled because CHD is rare among individuals under 35 and no such patients presented during recruitment. Table 1 presents the demographic and disease-related characteristics of the participants. Two themes were identified for this study, including Struggling with Illness Identity and Long-Term Medication, Taking Medication as an Intruder in Life. The identified excerpts and analysis materials are provided in Supplementary Appendix S3. Full transcripts are not publicly available to protect participant privacy, as requested by the participants.

**Table 1:** Participant demographics and disease-related information.

No	Gender	Education^a^	Age (years)	Marital status	History of disease (Months)	Therapy class	AMI	Prescription^b^	Self-reported medication^c^	Occupation	Household registration	Insurance^d^
P1	Male	④	42	Single	30	Medical	No	ACD	AC	Factory worker	Urban	d
P2	Male	③	49	Married	24	Surgical	No	ACD	AC	White-collar employee	Urban	ab
P3	Male	②	51	Married	48	Medical	No	ABD	AB	Factory worker	Urban	d
P4	Male	②	39	Single	1	Surgical	No	ABCD	ABC	Taxi driver	Rural	a
P5	Male	⑤	49	Married	18	Medical	No	ABCD	ABC	White-collar employee	Urban	a
P6	Male	④	48	Single	84	Surgical	Yes	ABCD	None	Executive	Urban	bd
P7	Male	④	49	Married	34	Surgical	Yes	ABCD	None	Entrepreneur	Urban	c
P8	Female	⑥	55	Married	1	Medical	No	AD	AD	Teacher	Rural	d
P9	Male	②	49	Married	28	Surgical	Yes	ABCD	ABCD	Farmer	Rural	c
P10	Male	③	40	Married	1	Surgical	Yes	ACD	AC	Executive	Urban	bd
P11	Male	②	57	Married	1	Medical	No	ACD	None	Locksmith	Urban	a
P12	Male	③	53	Married	120	Surgical	Yes	ABCD	BC	Civil servant	Rural	d
P13	Male	③	55	Married	18	Medical	No	ABCD	AD	Truck driver	Rural	c
P14	Female	③	38	Married	8	Medical	No	ABCD	ABCD	Unemployed	Urban	a
P15	Male	②	47	Married	11	Surgical	Yes	ABCD	BCD	Construction Worker	Rural	a
P16	Male	②	58	Married	32	Medical	No	ABCD	BC	Civil servant	Urban	d
P17	Male	⑤	47	Married	13	Surgical	Yes	ACD	AC	White-collar Employee	Urban	c
P18	Female	②	45	Married	13	Surgical	No	ACD	CD	Unemployed	Urban	a
P19	Male	②	49	Married	56	Surgical	No	ABCD	None	Construction worker	Rural	c
P20	Female	③	46	Married	11	Medical	Yes	ACD	CD	Homemaker	Rural	a
P21	Male	③	44	Single	4	Surgical	Yes	ACD	AC	Unemployed	Rural	a
P22	Male	⑤	40	Single	1	Surgical	Yes	ABCD	ABD	White-collar Employee	Urban	d
P23	Male	⑥	49	Married	9	Surgical	Yes	AD	AD	Entrepreneur	Urban	bd
P24	Female	①	51	Single	1	Medical	No	ACD	CD	Farmer	Rural	a
P25	Male	⑤	35	Single	1	Surgical	Yes	ABCD	ABC	Teacher	Urban	d
P26	Female	①	57	Married	44	Surgical	No	ABD	ABD	Homemaker	Rural	a
P27	Female	②	57	Married	76	Medical	No	ACD	D	Executive	Urban	d

^a^ ①Elementary school or less; ②Middle school; ③High school; ④Associate degree; ⑤Bachelor's degree; ⑥Master's Degree or more.

^b^ Discharge regimen (retrieved from the hospital's electronic medical records prior to each interview). A: antiplatelet agents; B: renin–angiotensin–aldosterone system inhibitors; C: beta-blockers; D: hypolipidemic agents.

^c^ Current regimen at interview (medications reported as taken in the past 7 days, cross-checked against EMR during the interview).

^d^ a: Urban–Rural resident basic medical insurance; b: commercial insurance; c: self-pay; d: Urban employee basic medical insurance; ab: Urban-Rural resident basic medical insurance + commercial insurance; bd: commercial insurance + Urban employee basic medical insurance. For ad insurance, there is a reimbursement conflict, so only one option can be selected initially.

### Theme 1. Struggling with illness identity and long-term medication

Young and middle-aged adults struggled to accept the role of patients. They usually viewed CHD as an “acute” condition that could be cured after surgeries and short-term medication taking. They lacked a clear understanding of the necessity of medications in CHD secondary prevention and regarded them merely as symptom relievers, which made them discontinue medications wrongly. In addition, they suffered from social isolation due to CHD and became pessimistic about CHD when receiving limited support from family and friends.

### Sub-theme 1. Viewing CHD as an acute condition

Many young and middle-aged adults believed CHD was an “acute” condition, which could be cured through surgeries and short-term medication taking. They had insufficient understanding about chronicity of CHD.

“I just really feel like I can turn this around. I've always believed my body can heal and I can get back to my old self completely”. (P8. 55 years old, female, newly diagnosed with CHD)

“I honestly think this isn't really a big deal. It's not even a real illness. It's just some clogged arteries, you get a surgery, and then you're back to being a normal, healthy person”. (P13. 55 years old, male, 1.5 years history of CHD)

They expected to fully recover through surgery and medication. However, viewing CHD as an acute condition, they were unprepared for its ongoing nature, perceiving disease progression as abnormal and feeling disappointed.

“I came to the hospital thinking the medication they gave me would cure the problem. How am I supposed to know if this medication works or not? Why did my condition flare up again not long after taking it?” (P10, 40 years old, male, newly diagnosed with CHD, AMI)

Some of them were confident that CHD could be cured because of their young age. Young age was regarded as an advantage in managing CHD, compared with old age. As a result, some of them undervalued the importance of adhering to medications.

“No matter what, I can always stay positive. We're still young, so I don't see this as a serious problem”. (P6. 48 years old, male, 7 years history of CHD, 2 AMIs)

Some young and middle-aged adults showed strong avoidance and denial toward the disease. They could not accept to live with CHD and take medications for the whole life, even queried the accuracy of the medical diagnosis. They regarded themselves as young and independent, rather than patients.

“I just can't wrap my head around it. Maybe the hospital was trying to make money or something, I really don't know. Most people only get symptoms when their arteries are like 80% blocked, right? But I'm still out working, fixing things and doing repairs, and I don't feel anything at all. I didn't really take the medicine seriously, and honestly, I didn't take the illness seriously either. I felt totally fine, you know? Sometimes I wonder if the doctor made a mistake or something. I just don't really believe it”. (P11. 57 years old, male, newly diagnosed with CHD)

Some participants, however, demonstrated a more accepting and reflective stance. Instead of denying the diagnosis, they tried to make sense of their condition in relation to their medical history and risk factors.

“At my age, women almost never get heart disease. It's kind of like the chance of getting into a car accident. From what I know, before 55 it's usually men, and it's really rare for women. So when I got it at 37, the odds were already super small. I’ve thought about it and maybe it's because I’ve had type 1 diabetes for so many years. For me, having CHD feels kind of expected, but for other people it might just be by chance. I’ve kept my diabetes under control for more than ten years with no problems, and then suddenly last year this came up. In a way, I was already prepared for it”. (P14, 38 years old, female, 1 year history of CHD)

Especially when comparing with their healthy peers, they expressed disappointment towards their role of CHD patients, which made them unwilling to adhere to medications.

“Taking medication all the time is really exhausting. I don't want to die, but if I keep feeling this way, maybe it's better to just give up. All my friends are healthy, and here I am, stuck with all these medications!” (P26. 57 years old, female, 4 years history of CHD)

“I used to be really happy, but then things changed. I was only forty back then, still pretty young, and out of nowhere I got diagnosed with heart disease and had to have surgery and take medication. My family's usually really healthy. My dad's 85 but acts like he's 60. My older brother's just a few years older than me, but when people see us, they say I look like I'm twenty years older than him. Having to take medications for so long at such a young age. Sometimes I feel like an old man”. (P12. 53 years old, male, 10 years history of CHD, AMI)

Viewing CHD as an acute illness, young and middle-aged adults often underestimated the importance of secondary prevention. Some of them viewed no symptoms as no CHD. They believed taking medications was a way to relieve symptoms, such as chest pain and tightness. one participant reported taking medications only when experiencing discomfort, reflecting a limited understanding of their necessity and long-term role in CHD management.

“I just took the medicine when symptoms came up occasionally. Later, when I didn't feel anything, I just let it go. I think I could handle it without the medications and control it myself”. (P19. 49 years old, male, 5 years history of CHD)

One young and middle-aged adult could not identify the benefits of taking medications, because his physical condition remained unchanged regardless of adherence. He was confused about the necessity of following the medication regimen while feeling asymptomatic, revealing that he did not fully understand how the medications worked or why they needed to be taken consistently.

“I'm not really sure if the medicine works. My body doesn't seem to react much, and honestly, I've never really felt uncomfortable anywhere”. (P23. 49 years old, male, 1.5 years history of CHD, AMI)

Some participants underestimated the chronic nature of CHD and the importance of medication. One young and middle-aged adults felt physically well and saw no clear impact on daily life, he thought missing a few doses was not a big deal.

“This illness doesn't really affect me, and my daily life isn't limited much. If I'm going out or on a short business trip, I just don't take the medications. Sometimes I forget, but it's no big deal”. (P16. 58 years old, male, 3 years history of CHD)

By comparison, young and middle-aged adults who experienced frequent symptoms or had a history of myocardial infarction placed greater importance on taking medication.

“I didn't feel anything unusual before. Honestly, I just didn't take it seriously, so I stopped taking my medication and kept smoking. Then one day I broke out in a heavy sweat and had chest pain, so I went to the hospital. After a blood test, the doctor told me I had to be admitted to the ICU that same night. That's when I realized how bad things really were. I read the report myself, it was an ST-segment elevation myocardial infarction. They even issued a critical condition notice to my family. The doctor stayed late to perform the surgery that night. That whole experience really stuck with me. I knew I couldn't keep brushing it off. Skipping my medication had serious consequences”. (P3. 51 years old, male, 4 years history of CHD)

### Sub-theme 2. Suffering from social isolation due to CHD

Some young and middle-aged adults felt ashamed of their identity as CHD patients and feared discrimination or judgment from others. As a result, they avoided discussing their disease and treatment. They tended to view medication-taking as a private matter, chose to hide or avoid taking medication.

“Usually, I don't talk about it. Taking my medication is personal. Why would I tell people I'm on them? How would that make people look at me?” (P3. 51 years old, male, 4 years history of CHD)

“I usually try to hide or avoid taking my medications around other people. Partly because I feel it makes me look weak. Also, I don't want others to see me differently. You can never really tell what people are thinking. Some might feel sorry for me, some might not care, and some might even enjoy seeing me struggle. So why cause myself that kind of trouble?” (P17. 47 years old, male, AMI)

One participant said they did not want others to know about their illness or medication use, as it felt like seeking sympathy. This touched on their sense of self-respect.

“Telling others you're sick and on medication feels like you're asking for their pity, I really don't want that. I'd rather come across as strong”. (P4. 39 years old, male, newly diagnosed with CHD)

Due to the need to follow strict medication routines, they had difficulty in fitting in friends. They worry that others avoid them due to concerns about their illness. To avoid rejection, some participants reported taking their medication in secret, delaying doses, or skipping them during social gatherings.

“Sometimes people don't want to hang out with you once they know you have this illness. When we're together, others might worry about what would happen if something goes wrong with you because they don't want to be responsible. So they tend to avoid you. Sometimes when I need to take my meds, I wait until they leave before taking them or just skip it this time”. (P27. 57 years old, female, 6 years history of CHD)

“They don't ask you to join them for meals anymore. You can't be like them. They can drink, but you have to take medication and can't drink. You just sit there watching them brag after drinking, and it really gets on my nerves”. (P7. 49 years old, male, 3 years history of CHD, AMI)

Some of them expected support from others, such as psychological support and reminding them take medications. Nevertheless, some young and middle-aged adults were concerned about being a burden of others, due to their unstable health condition.

“I've always handled my meds myself. Sometimes when I'm in a hurry in the morning, I might miss a dose. It'd be cool if someone reminded me now and then, haha”. (P25. 35 years old, male, newly diagnosed with CHD, AMI)

“No one has ever paid much attention to whether I take my medication or not. My husband and daughter are both just…” (she lowers her head and goes quiet) (P27. 57 years old, female, 6 years history of CHD)

“If you have this illness, then don't be a burden to others. Just handle it yourself, right? Why keep bothering people? It's tough for them and for you too. You're not that old, you should still try to stay strong”. (P10. 40 years old, male, newly diagnosed with CHD, AMI)

### Theme 2. Taking medication as an intruder in life

Medication taking was regarded as a barrier in the normal life of middle and young aged adults. Adhering to medications interrupted their life routine and made it difficult for them to fulfill personal social responsibility.

### Sub-theme 1. Disrupting daily routines due to medications

After being diagnosed with CHD, young and middle- aged adults struggled in establishing the medication taking habit. Especially when they were occupied with work, limited time was available for them to get prescriptions, as it often required taking time off from work. Some of them complained that getting prescriptions was time consuming. A participant even reduced the dosage to prolong the intervals of getting prescriptions for convenience.

“Sometimes, when the timing is tight, I have to take time off from work just to get a refill. If I can't go myself, I have to ask someone in the family to do it. It's honestly a hassle”. (P3, 51 years old, male, 4 years history of CHD)

“Every time I get my medication, I can only get a month's supply. I've estimated that if I reduce the dosage, it should last almost two months. So, the overall impact of this way of taking the medication is negligible, which is why I've decided to cut the dosage in half”. (P6. 48 years old, male, 7 years history of CHD, 2AMIs)

After being diagnosed with CHD, young and middle-aged adults struggled in establishing the medication taking habit. Two participant always forgot to take medications due to the busy working schedule.

“I usually never miss my morning meds since I take them right after I wake up. It's the statin at night that I sometimes forget. Like if I get home late after work or my routine gets thrown off, I might miss a dose once in a while”. (P1. 42 years old, male, 2 years history of CHD)

“I'm only on two meds, just started like a month ago. Sometimes I forget the statin at night. I'll come home, wash up, brush my teeth, go straight to bed, and then the next day I'm like, oh, I didn't take it! Maybe after a while I'll get into the habit like a reflex”. (P8, 55 years old, female, newly diagnosed with CHD)

Medication-related costs also became a burden. One participant explained that after their medical insurance funds were depleted, out-of-pocket expenses increased. These financial pressures often disrupted their regular medication use.

“My financial situation has gotten worse. Each time I buy my medication, every refill sets me back a few hundred yuan. There's no money left on my health insurance card now. I can't work, and I've used up all my savings”. (P9. 49 years old, male, 2 years history of CHD, AMI)

They experienced conflicts between medication taking and life routine. Adhering to medications was reported as decreasing the quality of life, because they had to follow the precautions required by medication taking, such as no drinking and smoking. Two participants would skip the dosage when he planned to drink.

“Taking all these meds, life just doesn't feel normal anymore. My quality of life has gone down. There are so many things I can't do my hobbies, drinking, all of it. You can't do this, can't eat that. What's the point of living like this? What is there left to do?” (P17. 47 years old, male, AMI)

“When I feel like having a drink, I know it's best not to mix it with my medication, but I still want to enjoy some light drinks, so I might skip my medication”. (P19. 49 years old, male, 5 years history of CHD)

Two young and middle-aged adults said that, although they wanted to take their medication as prescribed, they often had to disrupt their routine when traveling. One of them frequently traveled for work, and the other enjoyed taking long trips as a personal hobby. However, the limited amount of medication provided by the hospital was often insufficient for the entire duration of their trips.

“Taking medicine every day is a bit of a hassle. When I travel abroad for work, it's really inconvenient. I can only get one month's worth at a time, and the medicine overseas is different from what I take in China. It really makes things difficult”. (P21. 44 years old, male, 4 months history of CHD, AMI)

“I love traveling. Sometimes I go to places like Japan or the States, and my son lives abroad so I want to visit him. When I go, I usually stay for at least a month, but I can't always bring enough meds for the whole trip. It's not like I mean to skip them, it's just kind of out of my hands”. (P23. 49 years old, male, 9 months history of CHD, AMI)

### Sub-theme 2. Experiencing conflicts between medication taking and social responsibility

Young and middle-aged adults struggled to balance the CHD management and personal development. They needed to put forth effort in both their disease management and their work. Sometimes, they prioritized work over taking their medication. Three young and middle-aged adults skipped doses when they were busy with work.

“If you want to grow and achieve more at work, the pressure just gets bigger. I'm not the type who wants to just coast through life. Sometimes we have to entertain clients in the evening, and it's already 9 or 10 by the time I get home, only then can I take my meds, or sometimes I just forget”. (P2. 49 years old, male, 2 years history of CHD)

“When I'm really busy, sometimes I just don't have time to take it. Sometimes, I'm rushing in the morning to get to work, and I still have to commute”. (P18. 45 years old, female, 1 year history of CHD)

“Sometimes I forget to take my meds. Morning, noon, or night …… I forget pretty often. I'm busy during the day. I drive a taxi, so I'm on the road all the time. By the time I get home late at night, I'm just exhausted and it slips my mind”. (P4. 39 years old, male, newly diagnosed with CHD)

In traditional Asian societies, women may face challenges in medication adherence due to the combined demands of employment and household responsibilities. Two young women mentioned that, in addition to their job, they were so occupied with hosting guests and managing household chores that they occasionally forgot to take their medication.

“I've got a kid in school to look after. Sometimes when guests come over, I get caught up in hosting and then I realize I didn't take my medication”. (P20. 46 years old, female, 1 year history of CHD, AMI)

“I get home and still have to take care of four kids, which is exhausting. Washing clothes, mopping floors, and doing chores for hours. The kids are only seven or eight years old so they are too young to help out. It feels like the housework never ends because there is always something else to do. Sometimes I get so tired I just want to quit my job and go back to my hometown. I'm worn out and there is always something to do every day. When I'm done, if I remember I take my medication but if I don't there is nothing I can do about it”. (P26. 57 years old, female, 4 years history of CHD)

Another young and middle-aged adult expressed concern that having a chronic illness at a young age made him less competitive in the job market. He believed that employers would prefer healthy candidates and that someone with a recent serious illness and long-term medication needs would be at a disadvantage.

“Getting something like this at a young age puts you at a disadvantage. You're already losing points in areas where you should be able to work hard and fight for something. For example, if a boss has to choose between two people, one who just recovered from a serious illness and takes medicine every day, and one who is completely healthy, who do you think they would choose?” (P22. 40 years old, male, newly diagnosed with CHD, AMI)

A young and middle-aged adult said he was paying off a mortgage and car loan while supporting his family. He had planned to work hard while he was still young in order to build a better future for his wife and child. However, the doctor's advice to rest for a year and take lifelong medication left him feeling anxious and uncertain.

“I'm carrying loans for a house and a car and still have to support my family. If I get replaced at work now, it's really hard to bounce back. At this point, I'm already under a lot of pressure at home, thinking I should push myself while I'm still young to build a better future for my family and kids. Then suddenly something like this happens, and the doctor tells you to rest for a year and take meds for life. Honestly, I'm really at a loss”. (P22. 40 years old, male, newly diagnosed with CHD, AMI)

In contrast to the negative attitudes and poor medication adherence seen in some young and middle-aged adults, someone had a more positive view of medication. Following medical advice and taking their medication properly was even more important, so as not to create difficulties for their loved ones or society.

“Once you're in society, you have to take responsibility. At our age, we're stuck between looking after our parents and raising our kids. They all depend on us. My son once asked me, “Dad, are you worried?” I said, “If it weren't for you guys, what would I even worry about?” If you want to take care of your family, you've got to stay healthy first. That's just how it is. Either you live or you don't. And if you're still alive, then you've got to do your best to live well. So yeah, of course, I need to take my medication properly”. (P5. 49 years old, male, 1.5 years history of CHD)

“With heart disease, you really have to rely on yourself. Living healthy matters, and taking the meds is the most important part. In the end, it's your responsibility. You want to stay well so you don’t drag your family down. If your health gets worse, it's a burden on them. Only when you look after yourself can you take care of your family”. (P14, 38 years old, female, 1 year history of CHD)

## Discussion

This study explored the challenges of maintaining long-term medication adherence among young and middle-aged adults with CHD. We found that many young and middle-aged adults had distorted illness perceptions. They struggled to accept their condition as chronic and doubted the necessity of long-term medication. Besides, medication-taking often held a low priority in their daily lives.

Young and middle-aged adults with CHD misunderstood the nature of the disease. They often viewed surgery or short-term medication as a cure, leading them to discontinue treatment once symptoms subsided. Age-related optimism and strong confidence in physical recovery also played a role. Relying on medication was seen as a sign of aging or weakness. According to the Common-Sense Model of Self-Regulation (48), their misunderstanding of the illness led them to believe that long-term medication was unnecessary.

Many of them also had difficulty accepting the reality of living with a chronic illness. Charmaz noted that people with chronic illnesses must accept their condition before they can move forward (49). For them, taking daily medication symbolized being sick. This conflicted with their self-image as being young, capable, and independent individuals (50). Avoiding medication was a subtle way to reject the chronic, irreversible nature of CHD and to preserve a sense of being “just like everyone else”.

This emotional resistance was stronger in social situations. Previous studies on young myocardial infarction survivors have shown that low energy can lead to social withdrawal (51). Our study revealed that comparisons with healthy peers, sensitivity to others' opinions and passive social exclusion can trigger intense feelings of shame and frustration. Some young and middle-aged adults skipped or hid their medication in public to protect themselves. Some only began reflecting on their lives and reevaluating what truly mattered after experiencing a serious health event, such as a heart attack. This led them to develop a new understanding of how to live with a chronic illness (52). These findings suggest that young and middle-aged adults with CHD who struggle with illness acceptance or show strong emotional resistance may benefit from additional psychological support as part of routine care. Acceptance and Commitment Therapy (ACT) is a context-based, transdiagnostic behavioral approach. The aim of ACT is to help patients face their thoughts and emotions with openness and acceptance. It also supports individuals recognize unhelpful behavior patterns and make adjustments (53, 54), which can help them maintain balance across important areas of life (55, 56). In a study of CHD patients aged 35–55, Sheibani found that ACT promoted acceptance-based coping with illness and treatment, increased the use of positive emotion regulation strategies, and reduced reliance on maladaptive ones (57). Building on this evidence, we hypothesize that ACT could help young and middle-aged adults with CHD connect medication use with their personal values, foster greater acceptance of their condition, and ultimately improve long-term adherence. However, the implementation of ACT may be constrained by nurse availability and patients’ busy schedules, which should be considered when designing interventions. Stepped-care approaches, such as brief ACT sessions or online ACT programs, may help overcome these issues (58, 59). Future research should explore these possibilities through nurse-led interventions.

Nevertheless, they still encounter practical barriers in daily life that hinder regular medication use. Their lives are often shaped by fast-paced schedules and overlapping responsibilities. As a result, establishing a consistent medication-taking regimen was challenging for many of them. Theories of time geography and rhythmanalysis offer valuable insights into how external factors can disrupt daily health behaviors, such as medication adherence (60, 61). Time geography suggests that all activities require specific time and space resources, and that daily tasks naturally compete for these limited resources (60, 62). Building on this idea and drawing from rhythmanalysis, McQuoid further distinguishes between space-time as a resource and space-time as rhythms, which helps to explain how young people balance paid work, daily responsibilities, and the management of chronic illness (63). Consistent with these theoretical perspectives, participants reported that limited time and space, along with rapidly changing daily routines, made it challenging to take their medication regularly. They had to divide their time and energy among work, family responsibilities, personal interests, and illness management. Overtime work, unexpected social events, business trips, and leisure travel further disrupted routines. Consequently, adherence often depended on memory rather than stable daily patterns. For those who facing such external barriers, interventions should prioritize making treatment easier to integrate into daily routines. Convenient prescription and delivery services or longer refill intervals can reduce these barriers (64), while linking medication with existing habits, such as waking up, eating, or going to bed, can promote habit formation and support consistent adherence (65). Digital tools, including mobile apps and electronic pillboxes, can provide efficient and autonomous support when used in nurse-led follow-ups. Evidence from patients with CHD and other cardiovascular conditions supports the benefits of these approaches (66–68). Young and middle-aged adults must balance disease management with demanding social responsibilities. Therefore, nurse-led digital interventions may be especially helpful for this group. In real-world settings, uptake may be limited by patients’ varying levels of digital literacy and the additional costs associated with implementing and accessing mobile health systems, which could affect feasibility and transferability. Simple, low-burden strategies, such as text message reminders prior to full mobile health programs, may help mitigate these challenges (69). However, evidence on the effectiveness of digital interventions for young and middle-aged adults with CHD remains limited, highlighting the need for randomized controlled trials to evaluate their outcomes.

While forgetting to take medication sometimes occurs, young and middle-aged adults also continually make decisions about what to prioritize. Managing CHD is just one of many responsibilities (70). According to role conflict theory (71), individuals who occupy multiple social roles cannot fulfill all role expectations at once. When conflicts arise, they must decide which role to prioritize. These decisions are often influenced by broader social norms and value systems. In China, striving is widely valued and is often reflected in family and workplace expectations (72–74). When it is internalized, young and middle-aged adults tend to emphasize personal achievement and conform to socially defined ideals of the “standard person”. Consequently, work and family responsibilities tend to be prioritized over long-term health management (50). Taking medication is often seen as a personal task that can be delayed, especially when it feels like an additional burden alongside other social obligations. To address this, interventions should reframe medication use so that it reflects cultural values of duty and self-care. Health education and public campaigns could present taking regular medication as a mean of achieving personal goals and meet daily obligations. Adherence would then be viewed not as a burden, but as a responsibility to oneself and one's family (50, 75). Framing adherence in this way may make interventions more culturally relevant and acceptable. It can also motivate patients to maintain long-term treatment. Patients can also be encouraged to join peer support groups led by a team of nurses, physicians, and pharmacists (76). Sharing experiences with others facing similar challenges provides both practical strategies and emotional reinforcement. This is often more motivating than traditional clinical advice (77). However, these findings have not yet been directly tested in young and middle-aged adults with CHD. We therefore hypothesize that nurse-led peer support interventions may be relevant for this group. Future mixed-methods studies or randomized controlled trials could be conducted to develop and evaluate such interventions, testing their feasibility, cultural acceptability, and long-term effectiveness.

## Implications

This study provides practical insights for nurses in Chinese hospitals and communities settings to enhance medication adherence among young and middle-aged adults with CHD. Nurses could improve their understanding about CHD and medication adherence through multiple approaches. Firstly, psychological support, such as Acceptance and Commitment Therapy, can help patients link medication use with personal values, enhance illness acceptance and psychological flexibility, and long-term adherence. To enhance feasibility in busy clinical settings, a stepped-care approach may be helpful. For example, brief ACT modules could be incorporated into routine nurse follow-ups, with each session focusing on a single core skill or strategy. Alternatively, online ACT programs could be offered to supplement nurse-led sessions. These approaches could help nurses save time, improve work efficiency, and provide patients with accessible psychological support without adding substantial workload. Secondly, busy schedules, multiple responsibilities, and irregular routines can create practical barriers of young and middle-aged adults with CHD. Nurses can play an active role in helping patients to integrate their medication into their daily routines. Digital tools, including mobile apps and electronic pillboxes, help patients manage medications efficiently and provide reminders, which may be especially useful for those balancing work and family responsibilities. Simple strategies such as text message reminders can be introduced before full adoption of mobile health programs to improve feasibility. When digital tools are available, nurses should provide brief guidance, introductory tutorials, or demonstration videos to help patients become familiar with and proficient in using them. Nurses can also advocate for or facilitate institutional and community initiatives, such as 60–90 day electronic prescription refills, synchronized medication schedules through nurse-pharmacist collaboration, and medication delivery services. Third, culturally tailored health education and public awareness initiatives can reframe adherence as a responsibility toward oneself and one's family, for example by emphasizing social values, using family-centered messaging, and highlighting relatable role models that connect medication use with responsibility and achievement. Fourth, peer support groups allow patients to share strategies for managing medication, cope with irregular schedules, and remember doses, while offering emotional encouragement and reducing isolation. Seeing others successfully manage similar challenges may strengthen patients' confidence and motivation to adhere to treatment. Finally, the effectiveness of these interventions needs to be tested in randomized controlled trials in Chinese hospitals and communities.

## Strengths and limitations

This study explored the factors influencing medication adherence in secondary prevention among young and middle-aged adults with CHD through in-depth interviews. Maximum variation sampling was used to enhance representativeness and transferability. The study also provided deeper insights into the psychological, emotional, and social dimensions related to adherence in this population. Importantly, the thematic analysis was grounded in participants' own expressions and real-life contexts, rather than being constrained by a pre-established theoretical framework. This approach strengthened the practical relevance and authenticity of the findings. However, this study has several limitations. Although some participants had rural household registration, representing rural patients attending tertiary hospitals, the single-center design may not capture challenges in other regions. In rural areas, distance and financial constraints may worsen medication access and reduce adherence (78, 79). Limited doctors and weak disease management systems in rural and primary care settings may also affect patient experiences (80, 81). In China, young and middle-aged adults with CHD often bypass primary care and go directly to tertiary hospitals compared with older patients (82, 83). Thus, tertiary hospital data are somewhat representative of this population, but selection bias remains. Occupational factors may also influence adherence, as job demands, flexibility, and autonomy affect medication management (84). The study also relied on participants' retrospective accounts, which may introduce recall bias. In addition, social desirability bias and power dynamics were possible given that the interviews were conducted by a nursing student in a care setting. Voluntariness, privacy, and the lack of impact on care were emphasized to mitigate these risks (see Supplementary Appendix S5, Reflexive Statement). Despite these limitations, the findings provide valuable insights into the challenges young and middle-aged adults with CHD face in urban tertiary hospitals. Future research should include large-scale, cross-regional quantitative surveys to validate these qualitative findings and enhance their robustness and applicability.

## Conclusion

This study found medication nonadherence among young and middle-aged adults with CHD may stem from distorted illness perceptions, emotional resistance to accepting a chronic condition, and the marginalization of medication-taking in the context of busy daily lives. These findings can help healthcare providers better understand how young and middle-aged adults view their illness and treatment, while also offering useful insights for developing more targeted and personalized clinical interventions.

## Data Availability

The original contributions presented in the study are included in the article/Supplementary Material, further inquiries can be directed to the corresponding author.

## References

[1] China Cardiovascular Health and Disease Report Compilation Group. Summary of China cardiovascular health and disease report 2022. Chin J Interv Cardiol. (2023) 31(07):485–508. 10.3969/j.issn.1000-3614.2023.06.001

[2] Collaborators GCoD. Global, regional, and national age-sex specific mortality for 264 causes of death, 1980–2016: a systematic analysis for the global burden of disease study 2016. Lancet. (2017) 390(10100):1151–210. 10.1016/S0140-6736(17)32152-9 28919116 PMC5605883

[3] World Health Organization. WHO reveals leading causes of death and disability worldwide: 2000–2019. (2020). Available online at: https://www.who.int/news/item/09-12-2020-who-reveals-leading-causes-of-death-and-disability-worldwide-2000-2019 (Accessed September 11, 2025).

[4] Chong B Jayabaskaran J Jauhari SM Chan SP Goh R Kueh MTW Global burden of cardiovascular diseases: projections from 2025 to 2050. Eur J Prev Cardiol. (2024) 32:1001–15. 10.1093/eurjpc/zwae281 39270739

[5] Meirhaeghe A Montaye M Biasch K Huo Yung Kai S Moitry M Amouyel P Coronary heart disease incidence still decreased between 2006 and 2014 in France, except in young age groups: results from the French MONICA registries. Eur J Prev Cardiol. (2020) 27(11):1178–86. 10.1177/2047487319899193 32098503

[6] Andersson C Vasan RS. Epidemiology of cardiovascular disease in young individuals. Nat Rev Cardiol. (2018) 15(4):230–40. 10.1038/nrcardio.2017.154 29022571

[7] Wu WY Berman AN Biery DW Blankstein R. Recent trends in acute myocardial infarction among the young. Curr Opin Cardiol. (2020) 35(5):524–30. 10.1097/HCO.0000000000000781 32694263 PMC8454219

[8] Martin SS Aday AW Allen NB Almarzooq ZI Anderson CAM Arora P 2025 heart disease and stroke statistics: a report of US and global data from the American heart association. Circulation. (2025) 151(8):e41–e660. 10.1161/cir.0000000000001303 39866113 PMC12256702

[9] Lei L Zhou HB Peng J Yang YZ. Epidemiological characteristics of acute myocardial infarction in Shenzhen residents from 2013 to 2014. Chronic Pathematol J. (2015) 16(05):486–9. 10.16440/j.cnki.1674-8166.2015.05.006

[10] Liu HX Gao W Zhao D Sun JY. Investigation on inpatient treatment of acute myocardial infarction in 13 Chinese and Western hospitals in Beijing. Chin J Cardiol. (2010) 38(4):306–10. 10.3760/cma.j.issn.0253-3758.2010.04.008 20654073

[11] Xu QX Wan QP Zhang X Xiong JJ Guo YW. Epidemiological characteristics of acute cardiovascular and cerebrovascular events in Jing'an District, Shanghai in 2018. China J Stroke. (2020) 15(07):753–8. 10.3969/j.issn.1673-5765.2020.07.010

[12] Wang X Gao M Zhou S Wang J Liu F Tian F Trend in young coronary artery disease in China from 2010 to 2014: a retrospective study of young patients ≤45. BMC Cardiovasc Disord. (2017) 17:1–8. 10.1186/s12872-016-0458-1 28061763 PMC5219759

[13] Wu P Yu S Wang J Zou S Yao D-S Xiaochen Y. Global burden, trends, and inequalities of ischemic heart disease among young adults from 1990 to 2019: a population-based study. Front Cardiovasc Med. (2023) 10:1274663. 10.3389/fcvm.2023.1274663 38075966 PMC10704897

[14] Azegami M Hongo M Yanagisawa S Yamazaki A Sakaguchi K Yazaki Y Characteristics of metabolic and lifestyle risk factors in young Japanese patients with coronary heart disease: a comparison with older patients. Int Heart J. (2006) 47(3):343–50. 10.1536/ihj.47.343 16823240

[15] Zimmerman FH Cameron A Fisher LD Ng G. Myocardial infarction in young adults: angiographic characterization, risk factors and prognosis (coronary artery surgery study registry). J Am Coll Cardiol. (1995) 26(3):654–61. 10.1016/0735-1097(95)00254-2 7642855

[16] Shah N Kelly A-M Cox N Wong C Soon K. Myocardial infarction in the “young": risk factors, presentation, management and prognosis. Heart Lung Circ. (2016) 25(10):955–60. 10.1016/j.hlc.2016.04.015 27265644

[17] Smith SC J Benjamin EJ Bonow RO Braun LT Creager MA Franklin BA AHA/ACCF secondary prevention and risk reduction therapy for patients with coronary and other atherosclerotic vascular disease: 2011 update: a guideline from the American heart association and American college of cardiology foundation. Circulation. (2011) 124(22):2458–73. 10.1161/CIR.0b013e318235eb4d 22052934

[18] Visseren FLJ Mach F Smulders YM Carballo D Koskinas KC Bäck M 2021 ESC guidelines on cardiovascular disease prevention in clinical practice. Eur Heart J. (2021) 42(34):3227–337. 10.1093/eurheartj/ehab484 34458905

[19] World Health Organization. Adherence to long-term therapies: evidence for action. (2003). Available online at: https://iris.who.int/handle/10665/42682 (Accessed September 11, 2025).

[20] Peter N Kochery R Raj T Parimalakrishnan S Selvamuthukumaran S. Non compliance and patient perceived problems in cardiovascular disease patients: a prospective study. Indo Am J Pharmaceutical Sci. (2017) 4(8):2249–54.

[21] Du L Cheng Z Zhang Y Li Y Mei D. The impact of medication adherence on clinical outcomes of coronary artery disease: a meta-analysis. Eur J Prev Cardiol. (2017) 24(9):962–70. 10.1177/2047487317695628 28436725

[22] Cutler RL Fernandez-Llimos F Frommer M Benrimoj C Garcia-Cardenas V. Economic impact of medication non-adherence by disease groups: a systematic review. BMJ Open. (2018) 8(1):e016982. 10.1136/bmjopen-2017-016982 29358417 PMC5780689

[23] Fischer AJ Feld J Lange SA Günster C Dröge P Engelbertz C Impact of guideline-directed drug therapy after ST-elevation myocardial infarction on outcome in young patients-age and sex-specific factors. J Clin Med. (2024) 13(13):3788. 10.3390/jcm13133788 38999354 PMC11242167

[24] Lip GYH Genaidy A Jones B Tran G Estes C Sloop S. Medication non-adherence patterns and profiles for patients with incident myocardial infarction: observations from a large multi-morbid US population. Eur J Clin Invest. (2023) 53(6):e13968. 10.1111/eci.13968 36789887

[25] Ho PM Bryson CL Rumsfeld JS. Medication adherence: its importance in cardiovascular outcomes. Circulation. (2009) 119(23):3028–35. 10.1161/CIRCULATIONAHA.108.768986 19528344

[26] Fournier JA Cabezón S Cayuela A Ballesteros SM Cortacero JA De La Llera LSD. Long-term prognosis of patients having acute myocardial infarction when≤ 40 years of age. Am J Cardiol. (2004) 94(8):989–92. 10.1016/j.amjcard.2004.06.051 15476609

[27] World Health Organization. Cardiovascular diseases. (2021). Available online at: https://www.who.int/zh/news-room/fact-sheets/detail/cardiovascular-diseases-(cvds) (Accessed September 11, 2025).

[28] Liu W Qian S Hu Y Zhang R. The serial mediation effects of social support and self-efficacy on health literacy and self-management behaviors among young and middle-aged cardiac patients after percutaneous coronary intervention: a cross-sectional study in China. Risk Manag Healthc Policy. (2024) 17:2893–906. 10.2147/rmhp.S486800 39600348 PMC11590655

[29] Wang DL. Status quo and influencing factors of self-management and self-efficacy of young and middle-aged patients after PCI in a city (master’s thesis). North China University of Science and Technology, Tangshan (2020).

[30] Xu WW. Medication adherence and its influencing factors among middle-aged and young patients with coronary heart disease (master’s thesis). Binzhou medical university, Yantai (2022).

[31] Zu LL Yuan H Wang LY. Current status and influencing factors of medication adherence for secondary prevention in middle-aged and young patients with myocardial infarction. Chin J Integr Nurs. (2022) 8(08):115–7. Available online at: https://www.nursing-tcwm.com/template/through/index

[32] Zhao XN Zhao LT. Social function and its influencing factors among young and middle-aged patients with coronary heart disease after returning to work following PCI. Nurs Res. (2022) 36(18):3328–32. 10.12102/j.issn.1009-6493.2022.18.025

[33] Lu J Cheng Z Chen X. Theoretical exploration of a youth development-oriented society in the process of high-quality population development. Youth Explor. (2024) 03:5–14. 10.13583/j.cnki.issn1004-3780.2024.03.001

[34] Smolderen KG Strait KM Dreyer RP D'Onofrio G Zhou S Lichtman JH Depressive symptoms in younger women and men with acute myocardial infarction: insights from the VIRGO study. J Am Heart Assoc. (2015) 4(4):e001424. 10.1161/jaha.114.001424 25836055 PMC4579927

[35] Xiong JM Su J Ke QQ Li YX Gong N Yang QH. Psychosocial adaptation profiles in young and middle-aged patients with acute myocardial infarction: a latent profile analysis. Eur J Cardiovasc Nurs. (2024) 23(3):267–77. 10.1093/eurjcn/zvad071 37503729

[36] Nicholas PK Breakey S White BP Brown MJ Fanuele J Starodub R Mental health impacts of climate change: perspectives for the ED clinician. J Emerg Nurs. (2020) 46(5):590–9. 10.1016/j.jen.2020.05.014 32828480 PMC7435280

[37] Waite F Diamond R Collett N Chadwick E Bold E Teale A-L The comments of voices on the appearance of patients with psychosis: ‘the voices tell me that I am ugly’. BJPsych Open. (2019) 5(5):e86. 10.1192/bjo.2019.66 31537204 PMC6788219

[38] Sandelowski M. Whatever happened to qualitative description? Res Nurs Health. (2000) 23(4):334–40. 10.1002/1098-240x(200008)23:4<334::aid-nur9>3.0.co;2-g 10940958

[39] Tong A Sainsbury P Craig J. Consolidated criteria for reporting qualitative research (COREQ): a 32-item checklist for interviews and focus groups. Int J Qual Health Care. (2007) 19(6):349–57. 10.1093/intqhc/mzm042 17872937

[40] World Health Organization. World report on ageing and health. (2015). Available online at: https://www.who.int/publications/i/item/9789241565042/ (Accessed September 11, 2025).

[41] National Bureau of Statistics of China. What are the most recent age ranges defined in China for youth, middle-aged, and elderly populations? Are there any other classifications? (2024). Available online at: https://www.stats.gov.cn/hd/lyzx/zxgk/202405/t20240524_1954105.html (Accessed September 11, 2025).

[42] World Health Organization. Ageing and health: World Health Organization. (2024). Available online at: https://www.who.int/news-room/fact-sheets/detail/ageing-and-health (Accessed September 11, 2025).

[43] Xing Y Yang X Liu X. Health-promoting lifestyle and its influencing factors among middle-aged and young patients with coronary heart disease after PCI. Contemp Nurse (Late Edit). (2023) 30(02):131–4. 10.19793/j.cnki.1006-6411.2023.06.036

[44] Johnson HM Warner RC LaMantia JN Bowers BJ. “I have to live like I'm old”. Young adults’ perspectives on managing hypertension: a multi-center qualitative study. BMC Fam Pract. (2016) 17:1–9. 10.1186/s12875-016-0428-9 26969619 PMC4788815

[45] Saunders B Sim J Kingstone T Baker S Waterfield J Bartlam B Saturation in qualitative research: exploring its conceptualization and operationalization. Qual Quant. (2018) 52(4):1893–907. 10.1007/s11135-017-0574-8 29937585 PMC5993836

[46] Braun V Clarke V. Using thematic analysis in psychology. Qual Res Psychol. (2006) 3(2):77–101. 10.1191/1478088706qp063oa

[47] Bradshaw C Atkinson S Doody O. Employing a qualitative description approach in health care research. Glob Qual Nurs Res. (2017) 4:2333393617742282. 10.1177/2333393617742282 29204457 PMC5703087

[48] Leventhal H Diefenbach M Leventhal EA. Illness cognition: using common sense to understand treatment adherence and affect cognition interactions. Cognit Ther Res. (1992) 16:143–63. 10.1007/BF01173486

[49] Charmaz K. Good Days, Bad Days: The Self in Chronic Illness and Time. New Jersey: Rutgers University Press (1991). p. 311.

[50] Yu HY Liu AK Qiu WY Su J Zhou XY Gong N ‘I'm still young…it doesn't matter’-A qualitative study on the neglect of prodromal myocardial infarction symptoms among young and middle-aged adults. J Adv Nurs. (2023) 79(1):332–42. 10.1111/jan.15474 36300715

[51] Andersson EK Borglin G Willman A. The experience of younger adults following myocardial infarction. Qual Health Res. (2013) 23(6):762–72. 10.1177/1049732313482049 23515297

[52] Baldacchino D. Myocardial infarction: a turning point in meaning in life over time. Br J Nurs. (2011) 20(2):107–14. 10.12968/bjon.2011.20.2.107 21278658

[53] Hayes SC Pierson H. Acceptance and commitment therapy. In: Arthur Freeman A Felgoise SH Nezu CM Nezu AM Reinecke MA, editors. Encyclopedia of Cognitive Behavior Therapy. MA: Springer-Verlag Press (2005). p. 1–4.

[54] Hayes SC Luoma JB Bond FW Masuda A Lillis J. Acceptance and commitment therapy: model, processes and outcomes. Behav Res Ther. (2006) 44(1):1–25. 10.1016/j.brat.2005.06.006 16300724

[55] Kashdan TB Rottenberg J. Psychological flexibility as a fundamental aspect of health. Clin Psychol Rev. (2010) 30(7):865–78. 10.1016/j.cpr.2010.03.001 21151705 PMC2998793

[56] Hayes SC Strosahl KD Wilson KG. Acceptance and Commitment Therapy: The Process and Practice of Mindful Change. North Carolina: Guilford Press (2011). p. 401.

[57] Sheibani H Sheibani KA Amreei NN Masrour MJ. An investigation of the effects of the acceptance and commitment therapy in groups on the cognitive strategies of emotion regulation and self-control in coronary heart disease patients. J Med Life. (2019) 12(4):361. 10.25122/jml-2019-0035 32025254 PMC6993306

[58] Ong CW Terry CL Levin ME Twohig MP. Examining the feasibility and effectiveness of online acceptance and commitment therapy self-help in a quasi-stepped care model: a pilot study. Psychol Serv. (2023) 20(1):166. 10.1037/ser0000596 34735201

[59] Spatola CA Rapelli G Giusti EM Cattivelli R Goodwin CL Pietrabissa G Effects of a brief intervention based on acceptance and commitment therapy versus usual care for cardiac rehabilitation patients with coronary heart disease (ACTonHEART): a randomised controlled trial. BMJ Open. (2024) 14(6):e084070. 10.1136/bmjopen-2024-084070 38866567 PMC11177674

[60] Hägerstrand T. What about people in regional science. In: de Boer E, editor. Transport Sociology: Social Aspects of Transport Planning. Oxford: Pergamon Press (1986). p. 143–58.

[61] Lefebvre H. Rhythmanalysis: Space, Time and Everyday Life. London: Bloomsbury Publishing (2013). p. 130.

[62] Shove E Trentmann F Wilk R. Time, Consumption and Everyday Life. London: Routledge (2020). p. 250.

[63] McQuoid J Welsh J Strazdins L Griffin AL Banwell C. Integrating paid work and chronic illness in daily life: a space-time approach to understanding the challenges. Health Place. (2015) 34:83–91. 10.1016/j.healthplace.2015.04.001 25968586

[64] Wen G Zare H Eisenberg MD Anderson G. Association between non-profit hospital community benefit spending and health outcomes. Health Serv Res. (2023) 58(1):107–15. 10.1111/1475-6773.14060 36056796 PMC9836951

[65] Visscher BB Steunenberg B Heerdink ER Rademakers J. Medication self-management support for people with diabetes and low health literacy: a needs assessment. PLoS One. (2020) 15(4):e0232022. 10.1371/journal.pone.0232022 32330161 PMC7182204

[66] Ni Z Wu B Yang Q Yan LL Liu C Shaw RJ. An mHealth intervention to improve medication adherence and health outcomes among patients with coronary heart disease: randomized controlled trial. J Med Internet Res. (2022) 24(3):e27202. 10.2196/27202 35262490 PMC8943565

[67] Zullig LL Ramos K Bosworth HB. Improving medication adherence in coronary heart disease. Curr Cardiol Rep. (2017) 19(11):113. 10.1007/s11886-017-0918-y 28940020

[68] Nelson AJ Pagidipati NJ Bosworth HB. Improving medication adherence in cardiovascular disease. Nat Rev Cardiol. (2024) 21(6):417–29. 10.1038/s41569-023-00972-1 38172243

[69] Park LG Howie-Esquivel J Chung ML Dracup K. A text messaging intervention to promote medication adherence for patients with coronary heart disease: a randomized controlled trial. Patient Educ Couns. (2014) 94(2):261–8. 10.1002/14651858.CD011851.pub3 24321403

[70] Strauss AL. Chronic illness and the quality of life. AJN Am J Nurs. (1976) 76:82. In Carl A, editor press. Available online at: https://journals.lww.com/ajnonline/citation/1976/01000/CHRONIC_ILLNESS_AND_THE_QUALIT

[71] Goode WJ. A theory of role strain. Am Sociol Rev. (1960) 25:483–96. 10.2307/2092933

[72] Hu J Wang D. Actual personality and ideal personality of adolescents’ self-report and their parental expectation of them. Chin J Clin Psychol. (2009) 17(5):601–4. Available online at: http://clinicalpsychojournal.com/Magazine/Show.aspx?ID=139898

[73] Wang B Yu H. An analysis of the “996” phenomenon from the perspective of Marxist concept of labor: on the relationship between labor and development in the new era. Future Devel. (2019) 43(07):7–11. 10.3969/j.issn.1003-0166.2019.07.002

[74] Wu LL Fan Q Chen C Zuo P. Problems and practical breakthroughs of labor education from the perspective of “activity-development”. China Educ Technol. (2025) 461(06):95-101-17. Available online at: https://zdjy.cbpt.cnki.net/portal/journal/portal/client/paper/8f35a6c73e198d0ce4b7f83f5441f8ee

[75] Kangasniemi M Hirjaba M Kohonen K Vellone E Moilanen T Pietilä AM. The cardiac patients’ perceptions of their responsibilities in adherence to care: a qualitative interview study. J Clin Nurs. (2017) 26(17–18):2583–92. 10.1111/jocn.13642 27862488

[76] Yi X Zhang Q Qi H Yan Q Peng X. The impact of nurse-led peer support interventions on psychological Status and quality of life after acute myocardial infarction stent implantation in China. J Cardiovasc Nurs. (2025) 10:1097. 10.1097/JCN.0000000000001247 PMC13064833 40834426

[77] Lin YY Yen WJ Hou WL Liao WC Lin ML. Mental health nurses’ tacit knowledge of strategies for improving medication adherence for schizophrenia: a qualitative study. Healthcare. (2022) 10(3):492. 10.3390/healthcare10030492 35326970 PMC8955025

[78] Wroth TH Pathman DE. Primary medication adherence in a rural population: the role of the patient-physician relationship and satisfaction with care. J Am Board Family Med. (2006) 19(5):478–86. 10.3122/jabfm.19.5.478 16951297

[79] Zhou Y. Construction of a predictive model of medication adherence among patients with coronary heart disease (master’s thesis). Hebei University, Tangshan (2018).

[80] Zhang SY. Discussion on the problems existing in human resource management of community hospitals. China Health Industry. (2019) 16(32):135–6. 10.16659/j.cnki.1672-5654.2019.32.135

[81] Zou G Zhang Z Walley J Gong W Yu Y Hu R Use of medications and lifestyles of hypertensive patients with high risk of cardiovascular disease in rural China. PLoS One. (2015) 10(5):e0124484. 10.1371/journal.pone.0124484 25932640 PMC4416726

[82] Zhang JL Li J Liang T Xu HL. Acceptance and satisfaction of chronic disease patients with community health management. Chin Primary Health Care. (2020) 34(08):71–3. 10.3969/j.issn.1001-568X.2020.08.0018

[83] Chen Y. Study on residents’ choice of medical service utilization based on community healthcare service level (master’s thesis). Shanghai University Of Engineering Science, Shanghai (2020).

[84] Kearney SM Aldridge AP Castle NG Peterson J Pringle JL. The association of job strain with medication adherence: is your job affecting your compliance with a prescribed medication regimen? J Occup Environ Med. (2016) 58(7):707–11. 10.1097/JOM.0000000000000733 27206122

