# Supplementary material for: Exploring challenges to medication adherence aacmong young and middle-aged adults with coronary heart disease in China: a qualitative study
Source: Front Cardiovasc Med. 2025 Oct 30;12:1664013. doi: 10.3389/fcvm.2025.1664013 (PMC12611870; doi:10.3389/fcvm.2025.1664013)
Supplement: Supplementary file 1 [file Datasheet1.docx]

**Appendix 1** Consolidated criteria for reporting qualitative studies (COREQ): 32-item checklist

| **Item No.** | **Topic** | **Guide Questions/Description** | **Reported in this study** |
| --- | --- | --- | --- |
| **Domain 1: Research team and reflexivity** | | | |
| ***Personal Characteristics*** | | | |
| 1 | Interviewer/facilitator | Which author/s conducted the interview? | The first author |
| 2 | Credentials | What were the researcher’s credentials? | Postgraduate student in Nursing,  Registered Nurse (RN) |
| 3 | Occupation | What was their occupation at the time of the study? | Registered Nurse |
| 4 | Gender | Was the researcher male or female? | Female |
| 5 | Experience and training | What experience or training did the researcher have? | Trained in qualitative research |
| ***Relationship with participants*** | | | |
| 6 | Relationship established | Was a relationship established prior to study commencement? | Yes |
| 7 | Participant knowledge of the interviewer | What did the participants know about the researcher? e.g., personal goals, reasons for doing the research | Participants were informed of the purpose and reasons for doing the research |
| 8 | Interviewer characteristics | What characteristics were reported about the interviewer/facilitator? e.g. Bias, assumptions, reasons and interests in the research topic | The interviewer remained impartial, objective, and nonjudgmental |
| **Domain 2: study design** | | | |
| ***Theoretical framework*** | | | |
| 9 | Methodological orientation and Theory | What methodological orientation was stated to underpin the study? e.g. grounded theory, discourse analysis, ethnography, phenomenology, content analysis | Descriptive research  Thematic Analysis |
| ***Participant selection*** | | | |
| 10 | Sampling | How were participants selected? e.g. purposive, convenience, consecutive, snowball | Purposive |
| 11 | Method of approach | How were participants approached? e.g. face-to-face, telephone, mail, email | Face to Face |
| 12 | Sample size | How many participants were in the study? | Twenty-seven |
| 13 | Non-participation | How many people refused to participate or dropped out? Reasons? | None |
| ***Setting*** | | | |
| 14 | Setting of data collection | Where was the data collected? e.g. home, clinic, workplace | Private room |
| 15 | Presence of non-participants | Was anyone else present besides the participants and researchers? | No |
| 16 | Description of sample | What are the important characteristics of the sample? e.g. demographic data, date | Demographics and disease-related information |
| ***Data collection*** | | | |
| 17 | Interview guide | Were questions, prompts, guides provided by the authors? Was it pilot tested? | Interview guideline provided by the research team and pre-interviews were conducted by the first author and guided by the last author |
| 18 | Repeat interviews | Were repeat interviews carried out? If yes, how many? | No |
| 19 | Audio/visual recording | Did the research use audio or visual recording to collect the data? | The research used audio recording to collect the data |
| 20 | Field notes | Were field notes made during and/or after the interview? | Yes |
| 21 | Duration | What was the duration of the interviews? | 30 to 90 minutes |
| 22 | Data saturation | Was data saturation discussed? | Yes |
| 23 | Transcripts returned | Were transcripts returned to participants for comment and/or correction? | Yes |
| **Domain 3: analysis and findings** | | | |
| ***Data analysis*** | | | |
| 24 | Number of data coders | How many data coders coded the data? | Two |
| 25 | Description of the coding tree | Did authors provide a description of the coding tree? | Yes |
| 26 | Derivation of themes | Were themes identified in advance or derived from the data? | Derived from the data |
| 27 | Software | What software, if applicable, was used to manage the data? | MAXQDA 2020 |
| 28 | Participant checking | Did participants provide feedback on the findings? | Yes |
| ***Reporting*** | | | |
| 29 | Quotations presented | Were participant quotations presented to illustrate the themes / findings? Was each quotation identified? e.g. participant number | Yes |
| 30 | Data and findings consistent | Was there consistency between the data presented and the findings? | Yes |
| 31 | Clarity of major themes | Were major themes clearly presented in the findings? | Yes |
| 32 | Clarity of minor themes | Is there a description of diverse cases or discussion of minor themes? | Yes |

**Appendix 2** Interview guide

Hi, my name is Jianli Guo, and I’m a nursing graduate student from Shanghai Jiao Tong University School of Medicine. How are you feeling today? I’d like to talk with you about your experiences and thoughts related to taking medications for coronary heart disease. This might take a bit of your time, and I really appreciate you being here. Please feel free to share anything you’re comfortable talking about。

The finalized interview guide included the following main questions:

1. Since your last discharge, how have you been taking your medications throughout the day? (name of the drug, side effects, pharmacological effects, dosage, time of intake)
2. What barriers have you encountered in taking your medications?
3. When you take your medications, is there usually someone who helps or reminds you? (Who usually helps you, and in what ways do they assist or remind you to take your medications?)
4. What are your thoughts or feelings about your illness? (Understanding of the illness, Emotional response to the disease)

**Appendix 3** The main data analysis process

| **Theme** | **Subtheme** | **Codes** | **Representative quote** |
| --- | --- | --- | --- |
| **Struggling with Illness Identity and Long-Term Medication** | **Viewing CHD as an acute condition** | Limited understanding of disease characteristics | "So you’re saying this medicine works like it gets rid of the root cause of the disease, and then the disease is cured? Does that mean I can just stop taking the medicine after that?" **(P16, 58 years old, male, 32 months history of CHD, no AMI)**  "I don’t really understand this disease. It’s an acute illness, right?" **(P27, 57 years old, female, 76 months hostory of CHD, no AMI)** |
|  |  | Perceive surgery as a definitive solution | “The surgery’s done, and my wounds have all healed. Everything seems fine, and I don’t feel like there’s anything wrong with me.” **(P4, 39 years old, male, newly diagnosed with CHD, no AMI)**  "I honestly think this isn’t really a big deal. it's not even a real illness. It’s just some clogged arteries, you get a surgery, and then you’re back to being a normal, healthy person." **(P13, 55 years old, male, 18 months hostory of CHD, no AMI)** |
|  |  | Expectation of disease reversibility | "I just really feel like I can turn this around. I’ve always believed my body can heal and I can get back to my old self completely." **(P8, 55 years old, female, newly diagnosed with CHD, no AMI)** |
|  |  | Relapse seen as abnormal | "I came to the hospital thinking the medication they gave me would cure the problem. How am I supposed to know if this medication works or not? Why did my condition flare up again not long after taking it?" **(P10, 40 years old, male, newly diagnosed with CHD, AMI)** |
|  |  | Underestimate the necessity of medication | "I just took the medicine when symptoms came up occasionally. Later, when I didn’t feel anything, I just let it go. I think I could handle it without the medications and control it myself." **(P19, 49 years old, male, 56 months hostory of CHD, no AMI)** |
|  |  | Young and confident in disease coping | "No matter what, I can always stay positive. We’re still young, so I don’t see this as a serious problem." **(P6, 48 years old, male, 84 months hostory of CHD, 2 AMIs)**    “Sometimes I think I’m still pretty young, so it’s okay if I occasionally forget or miss a dose.” **(P27, 57 years old, female, 76 months hostory of CHD, no AMI)** |
|  |  | Life goes on as usual | "This illness doesn’t really affect me, and my daily life isn’t limited much. If I’m going out or on a short business trip, I just don’t take the medications. Sometimes I forget, but it’s no big deal." **(P16, 58 years old, male, 76 months hostory of CHD, no AMI)**  "I didn’t feel anything unusual before. Honestly, I just didn’t take it seriously, so I stopped taking my medication and kept smoking. Then one day I broke out in a heavy sweat and had chest pain, so I went to the hospital. After a blood test, the doctor told me I had to be admitted to the ICU that same night. That’s when I realized how bad things really were. I read the report myself. it was an ST-segment elevation myocardial infarction. They even issued a critical condition notice to my family. The doctor stayed late to perform the surgery that night. That whole experience really stuck with me. I knew I couldn’t keep brushing it off. Skipping my medication had serious consequences." **(P3, 51 years old, male, 48 months hostory of CHD, no AMI)** |
|  |  | Low perceived efficacy of medication | "I’m not really sure if the medicine works. My body doesn’t seem to react much, and honestly, I’ve never really felt uncomfortable anywhere." **(P23, 50 years old, male, 9 months history of CHD, AMI)** |
|  |  | Denial of disease | "I just can’t wrap my head around it. Maybe the hospital was trying to make money or something, I really don’t know. Most people only get symptoms when their arteries are like 80% blocked, right? But I’m still out working, fixing things and doing repairs, and I don’t feel anything at all. I didn’t really take the medicine seriously, and honestly, I didn’t take the illness seriously either. I felt totally fine, you know? Sometimes I wonder if the doctor made a mistake or something. I just don’t really believe it." **(P11, 57 years old, male, newly diagnosed with CHD, no AMI)**  "How am I supposed to see it? I just can’t. I’ve never really been able to face it. I didn’t take it seriously in the first place." **(P7, 49 years old, male, 34 months history of CHD, AMI)**  "What’s the use of not accepting being sick? It’s pointless! There’s nothing else you can do anyway. What can you do? You’re not a miracle worker." **(P24, 51 years old, female, newly diagnosed with CHD, no AMI)**  "At my age, women almost never get heart disease. It’s kind of like the chance of getting into a car accident. From what I know, before 55 it’s usually men, and it’s really rare for women. So when I got it at 37, the odds were already super small. I’ve thought about it and maybe it’s because I’ve had type 1 diabetes for so many years. For me, having CHD feels kind of expected, but for other people it might just be by chance. I’ve kept my diabetes under control for more than ten years with no problems, and then suddenly last year this came up. In a way, I was already prepared for it." **(P14, 38 years old, female, 12 months history of CHD, no AMI)** |
|  |  | Inferiority compared to healthy peers | "Taking medications all the time is really exhausting. I don’t want to die, but if I keep feeling this way, maybe it’s better to just give up. All my friends are healthy, and here I am, stuck with all these medications!" **(P26, 57 years old, female, 44 months history of CHD, no AMI)**  "I used to be really happy, but then things changed. I was only forty back then, still pretty young, and out of nowhere I got diagnosed with heart disease and had to have surgery and take medication. My family’s usually really healthy. My dad’s 85 but acts like he’s 60. My older brother’s just a few years older than me, but when people see us, they say I look like I’m twenty years older than him. Having to take medications for so long at such a young age. Sometimes I feel like an old man." **(P12, 53 years old, male, 120 months history of CHD, AMI)** |
|  | **Suffering from social isolation due to CHD** | Friends distancing themselves | "Sometimes people don’t want to hang out with you once they know you have this illness. When we’re together, others might worry about what would happen if something goes wrong with you because they don’t want to be responsible. So they tend to avoid you. Sometimes when I need to take my meds, I wait until they leave before taking them or just skip it this time."  **(P27, 57 years old, female, 76 months hostory of CHD, no AMI)**  P7: "They don’t ask you to join them for meals anymore. You can’t be like them. They can drink, but you have to take medication and can’t drink. You just sit there watching them brag after drinking, and it really gets on my nerves." **(P7, 49 years old, male, 34 months history of CHD, AMI)** |
|  |  | Lacking of social support | "I’ve always handled my meds myself. Sometimes when I’m in a hurry in the morning, I might miss a dose. It’d be cool if someone reminded me now and then, haha.” **(P25, 35 years old, male, newly diagnosed with CHD, no AMI)**  "No one has ever paid much attention to whether I take my medication or not. My husband and daughter are both just..." (she lowers her head and goes quiet) **(P27, 57 years old, female, 76 months hostory of CHD, no AMI)** |
|  |  | Concerns about seeking help | "If you have this illness, then don’t be a burden to others. Just handle it yourself, right? Why keep bothering people? It’s tough for them and for you too. You’re not that old, you should still try to stay strong." **(P10, 40 years old, male, newly diagnosed with CHD, AMI)** |
|  |  | Medication as personal privacy | "Usually, I don’t talk about it. Taking my medication is personal. Why would I tell people I’m on them? How would that make people look at me?" **(P3, 51 years old, male, 48 months hostory of CHD, no AMI)** |
|  |  | Fear of stigma from medication | "I usually try to hide or avoid taking my medications around other people. Partly because I feel it makes me look weak. Also, I don’t want others to see me differently. You can never really tell what people are thinking. Some might feel sorry for me, some might not care, and some might even enjoy seeing me struggle. So why cause myself that kind of trouble?" **(P17, 47 years old, male, 13 months hostory of CHD, AMI)** |
|  |  | Self-esteem | "Telling others you’re sick and on medication feels like you’re asking for their pity, I really don’t want that. I’d rather come across as strong." **(P4, 39 tears old, male, newly diagnosed with CHD, no AMI)** |
| **Taking Medication as an Intruder in Life** | **Disrupting daily routines due to medications** | Time-consuming process of medication refills | “Every time I get my medication, I can only get a month's supply. I've estimated that if I reduce the dosage, it should last almost two months. So, the overall impact of this way of taking the medication is negligible, which is why I've decided to cut the dosage in half." **(P6, 48 years old, male, 84 months hostory of CHD, 2 AMIs)**  ”Sometimes, when the timing is tight, I have to take time off from work just to get a refill. If I can’t go myself, I have to ask someone in the family to do it. It’s honestly a hassle.” **(P3, 51 years old, male, 48 months hostory of CHD, no AMI)** |
|  |  | Financial burden of medication refills | "My financial situation has gotten worse. Each time I buy my medication, every refill sets me back a few hundred yuan. There’s no money left on my health insurance card now. I can’t work, and I’ve used up all my savings." **(P9, 49 years old, male, 28 months hostory of CHD, AMI)** |
|  |  | Conflict between meds and daily habits | "Taking all these meds, life just doesn’t feel normal anymore. My quality of life has gone down. There are so many things I can’t do my hobbies, drinking, all of it. You can’t do this, can’t eat that. What’s the point of living like this? What is there left to do?" **(P17, 47 years old, male, 13 months hostory of CHD, AMI)**  "When I feel like having a drink, I know it's best not to mix it with my medication, but I still want to enjoy some light drinks, so I might skip my medication."**(P19, 49 years old, male, 56 months hostory of CHD, no AMI)** |
|  |  | No habit of regular medication | "I usually never miss my morning meds since I take them right after I wake up. It’s the statin at night that I sometimes forget. Like if I get home late after work or my routine gets thrown off, I might miss a dose once in a while." **(P1, 42 years old, male, 30 months hostory of CHD, no AMI)**  “I’m only on two meds, just started like a month ago. Sometimes I forget the statin at night. I’ll come home, wash up, brush my teeth, go straight to bed, and then the next day I’m like, oh, I didn’t take it! Maybe after a while I’ll get into the habit like a reflex.” **(P8, 55 years old, female, newly diagnosed with CHD, no AMI**) |
|  |  | Insufficient medication supply during travel | "Taking medicine every day is a bit of a hassle. When I travel abroad for work, it’s really inconvenient. I can only get one month’s worth at a time, and the medicine overseas is different from what I take in China. It really makes things difficult." **(P21, 44 years old, male, 4 months hostory of CHD, AMI)**  "I love traveling. Sometimes I go to places like Japan or the States, and my son lives abroad so I want to visit him. When I go, I usually stay for at least a month, but I can't always bring enough meds for the whole trip. It’s not like I mean to skip them, it’s just kind of out of my hands." **(P23, 49 years old, male, 9 months hostory of CHD, AMI)** |
|  | **Experiencing conflicts between medication taking and social responsibility** | Busy with career advancement | “If you want to grow and achieve more at work, the pressure just gets bigger. I’m not the type who wants to just coast through life. Sometimes we have to entertain clients in the evening, and it’s already 9 or 10 by the time I get home, only then can I take my meds, or sometimes I just forget." **(P2, 49 years old, male, 24 months hostory of CHD, no AMI)**  "When I’m really busy, sometimes I just don’t have time to take it. Sometimes, I’m rushing in the morning to get to work, and I still have to commute." **(P18, 45 years old, female, 13 months hostory of CHD, no AMI)**  "Sometimes I forget to take my meds. Morning, noon, or night......I forget pretty often. I’m busy during the day. I drive a taxi, so I’m on the road all the time. By the time I get home late at night, I’m just exhausted and it slips my mind." **(P4, 39 years old, male, newly diagnosed with CHD, no AMI)** |
|  |  | Medication missed due to caregiving and housework | "I’ve got a kid in school to look after. Sometimes when guests come over, I get caught up in hosting and then I realize I didn’t take my medication." **(P20, 46 years old, female, 11 months hostory of CHD, AMI)**  "I get home and still have to take care of four kids, which is exhausting. Washing clothes, mopping floors, and doing chores for hours. The kids are only seven or eight years old so they are too young to help out. It feels like the housework never ends because there is always something else to do. Sometimes I get so tired I just want to quit my job and go back to my hometown. I’m worn out and there is always something to do every day. When I’m done, if I remember I take my medication but if I don’t there is nothing I can do about it." **(P26, 57 years old, female, 44 months hostory of CHD, no AMI)**  “Sometimes, at our age, we start thinking about these things. We’re still young, with elderly parents to care for and young kids to raise. Taking medicine properly is the least we can do to avoid becoming a burden to our children. They’re still very young. Even if we can’t do much to help them, at least we shouldn’t make things harder for them.” **(P9, 49 years old, male, 28 months hostory of CHD, AMI)** |
|  |  | Decline in workplace competitiveness | "Getting something like this at a young age puts you at a disadvantage. You’re already losing points in areas where you should be able to work hard and fight for something. For example, if a boss has to choose between two people, one who just recovered from a serious illness and takes medicine every day, and one who is completely healthy, who do you think they would choose?" **(P22. 40 years old, male, newly diagnosed with CHD, AMI)** |
|  |  | Medication hinders return to work | “This illness actually affects getting back to work. you’re often expected to go out for socializing or entertain clients. In those moments, telling others you need to stop and sit aside to take your drugs just isn’t appropriate.” **(P22,40 years old, male, AMI)**  ”Sometimes, like when I want to find a job, I feel both the risk and the pressure. I see the money there, but I can’t earn it myself. it’s really frustrating.” **(P9, 49 years old, male, newly diagnosed with CHD, AMI)**  “This illness actually does affect going back to work. For example, when you're meeting with clients or showing them products, having to pause and say you need to take your medication doesn’t feel appropriate.” **(P22, 40 years old, male, newly diagnosed with CHD, AMI)** |
|  |  | Anxiety about having to take meds forever | "I’m carrying loans for a house and a car and still have to support my family. If I get replaced at work now, it’s really hard to bounce back. At this point, I’m already under a lot of pressure at home, thinking I should push myself while I’m still young to build a better future for my family and kids. Then suddenly something like this happens, and the doctor tells you to rest for a year and take meds for life. Honestly, I’m really at a loss." **(P22, 40 years old, male, newly diagnosed with CHD, AMI)**  "Once you're in society, you have to take responsibility. At our age, we’re stuck between looking after our parents and raising our kids. They all depend on us. My son once asked me, "Dad, are you worried?" I said, "If it weren’t for you guys, what would I even worry about?" If you want to take care of your family, you’ve got to stay healthy first. That’s just how it is. Either you live or you don’t. And if you’re still alive, then you’ve got to do your best to live well. So yeah, of course, I need to take my medication properly." **(P5, 49 years old, male, 18 months hostory of CHD, no AMI)**  “With heart disease, you really have to rely on yourself. Living healthy matters, and taking the meds is the most important part. In the end, it’s your responsibility. You want to stay well so you don’t drag your family down. If your health gets worse, it’s a burden on them. Only when you look after yourself can you take care of your family.”**(P14, 38 years old, female, 12 months hostory of CHD, no AMI)** |

**Appendix 4** Data structure figure


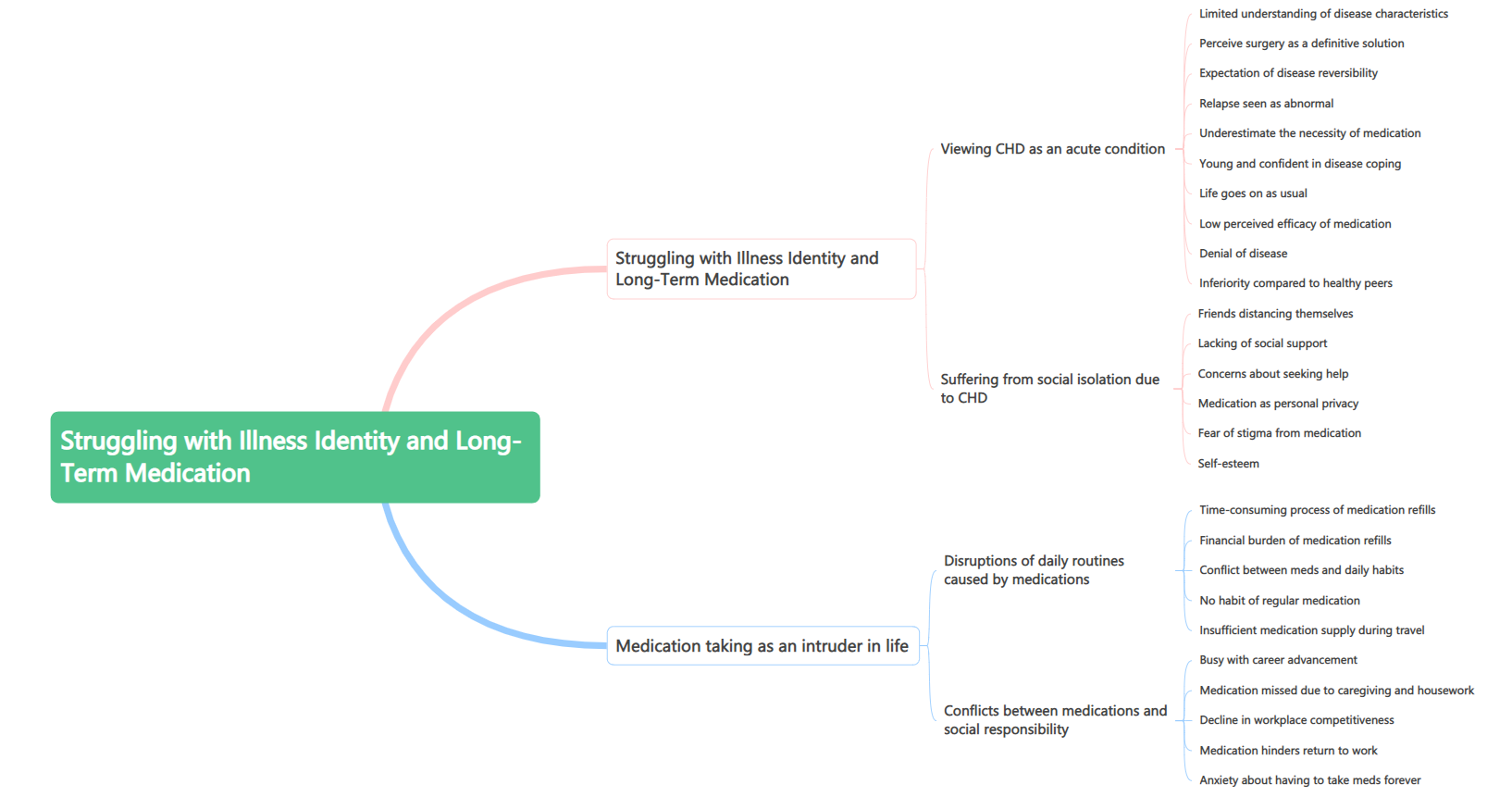


**Appendix 5** Reflexive statements

Personal reflexivity

| Data collection | I was the sole data collector in this study, which was also part of my master’s program. As a nursing graduate student with one year of clinical experience in a cardiology ward, I was already familiar with the research context and target population. This background led me to instinctively focus on issues such as patients’ work-related pressures for patients and their tendency to forget to take their medications. To prevent my personal experience and assumptions from limiting the scope of the interviews, I avoided imposing prior theoretical frameworks and instead adopting an open stance, allowing participants to articulate their perspectives as fully as possible and thereby enriching the data. In addition, some of my family members would have met the inclusion criteria, meaning encounters with participants in similar circumstances could evoke stronger empathy or bias. To address this, I undertook reflexive writing after interviews to acknowledge and manage these potential influences. As the interviews progressed and I heard the diverse insights of the participants, I found myself rethinking my own understanding of young and middle-aged patients with coronary heart disease. I also reflected on how I communicate with these patients in clinical practice. |
| --- | --- |
| Data analysis | Throughout the analysis, I remained acutely aware of how my background in nursing and experience in clinical cardiology could influence my interpretation of the data. Before beginning, I shared my transcriptions with participants for feedback, which helped minimize the risk of my own perspective shaping the recorded content. During coding, I found that my attention naturally gravitated towards factors that had been emphasised in previous research. However, I recognized that my younger target population might experience these issues differently. This prompted me to expand the open coding to capture all relevant information, including aspects not directly related to adherence. As I developed the codes into sub-themes and themes, I revisited the original recordings whenever I encountered ambiguous classifications. I repeatedly listened to the participants’ own words and reflecting on my preliminary assumptions to consider how they might shape my interpretation of the data. Team discussions further helped me identify points where my perspective could limit understanding. For example, my initial tendency to view adherence barriers as primarily knowledge gaps prompted me to expand my perspective to encompass identity conflicts and social pressures. This iterative process highlighted the strengths and limitations of my professional perspective, prompting me to consciously balance my clinical insights with the lived experiences of the participants. Engaging in this reflective practice refined the analysis and helped me recognise how the research process shaped my understanding of young and middle-aged patients with coronary heart disease. |
| Translation | While translating the participants’ quotations, I became aware of how my background could influence my decisions. when working on a narrative in which a participant repeatedly emphasised 'there is always something to do', I was instinctively tempted to remove the repetition, as it might sound redundant in English. However, on reflection, I realised that these repetitions were central to conveying the participant’s overwhelming fatigue and frustration, so I chose to preserve them in order to maintain the emotional intensity. I also noticed that my professional background led me to focus more on adherence behaviours than on the participants' feelings. Recognising these inclinations reminded me to prioritise participants' lived experiences and emotional meanings over my own stylistic or clinical preferences. This reflexive practice helped me balance linguistic precision with cultural and experiential fidelity. |

Interpersonal reflexivity

| Data collection | During the data collection process, I was mindful of the inherent power dynamics of my role. Despite my prior clinical experience as a nurse in cardiology, I introduced myself to participants primarily as a graduate nursing student rather than a practising nurse. This approach aimed to minimise the influence of my authority on participants’ responses and reduce the typical nurse–patient power differential. I also recognised that my position within the healthcare setting might lead participants to provide socially desirable responses, such as overstating their medication adherence or presenting themselves as responsible and compliant patients, in line with perceived clinical expectations. To mitigate this, I emphasised voluntariness and confidentiality at the beginning of each interview, clarified that participation would not affect their care, and adopted an open and nonjudgmental interviewing style to encourage honest disclosure. In addition, participants played an active role in shaping the research process. Their narratives shaped the topics I explored, the follow-up questions I asked and the depth of discussion in each interview. For instance, when a participant described feelings of social isolation due to their medication obligations, I adapted my follow-up questions to delve into the emotional impact of these experiences rather than directing the conversation towards clinical or adherence-related matters. Participants' perspectives therefore directly shaped both the data collected and my understanding of their lived experiences. I also reflected on how my personal experiences, including having family members who met the study inclusion criteria, could affect my reactions and empathy during interviews. When participants shared experiences similar to those of my family, I was careful not to allow extra empathy to bias the flow of questioning or the interpretation of their narratives. Through iterative reflection on interpersonal dynamics, I strove to balance my clinical perspective with the lived experiences of participants, thereby fostering trust and minimising the impact of inherent power differentials on the generated data. |
| --- | --- |
| Data analysis | During data analysis, I was mindful of how the interpersonal dynamics within the research team could influence my interpretation of the participants' accounts. After completing the initial coding and developing subthemes and themes, doctoral student Xu reviewed my work. Given Xu’s extensive research experience, I recognised that I might have been inclined to accept her suggestions without sufficient critical reflection. To address this, we discussed every coding adjustment in detail. Whenever we were uncertain, we returned to the original audio recordings to re-examine the participants' expressions and ensure that our interpretations remained grounded in their lived experiences. In cases where Xu and I could not reach consensus, we involved our supervisor, Zhu, in the discussion. Even then, rather than simply deferring to the judgement of more experienced team members, we referred back to the original recordings to verify participants’ intended meanings. This iterative process of discussion, verification and reflection helped to balance the influence of senior colleagues while maintaining fidelity to the voices of the participants. It also made me more aware of my tendency to align with perceived authority, prompting me to actively monitor and manage this tendency throughout the analysis. |
| Translation and back-translation | Throughout the translation and back-translation process, I remained aware of the interpersonal dynamics within the team and their potential influence on the final English quotations. Xu and I translated selected quotations into English independently, after which Zhu facilitated team discussions to reconcile differences and produce a harmonised version. As the less experienced translator, I recognised that I might be inclined to defer to the judgements of more senior team members, particularly given our prior agreement on philosophical categories and thematic structure. For instance, when translating the Chinese phrase “你又不是神仙啊”, I initially translated it literally as “You are not a god”, but Zhu and Xu suggested a more contextually appropriate English phrase, which I ultimately adopted. Yi Zhou, a native English speaker of Chinese descent who had not previously collaborated with the team, was engaged as a paid consultant to provide back-translations of the harmonised English quotations. When evaluating her suggestions, I consciously reflected on my potential biases and considered the power dynamics involved in balancing her outsider perspective with our clinical and research knowledge. All decisions, including those where we disagreed, were checked against the original audio recordings to ensure that the voices of the participants remained central. Engaging in these discussions and reflecting on my own position allowed me to recognise how interpersonal relationships within the team and with external collaborators could influence the translation process. |

Methodological reflexivity

| Data collection and analysis | We adopted a constructivist paradigm. Initially, one of the authors proposed using the broad theoretical framework of the Capability-Opportunity-Motivation-Behaviour (COM-B) model as a guide for analysis. However, after discussion, we decided against imposing a predetermined framework. As this is a qualitative study, our goal is to explore and understand the experiences of the participants rather than testing them against predefined expectations. We therefore conducted semi-structured interviews and analysed the data using inductive thematic analysis. This approach allowed us to remain open to the perspectives of young and middle-aged patients with coronary heart disease, free from the constraints of existing theories. As someone new to qualitative research, I aimed to improve the study’s rigour by reading methodological literature and completing formal training at my university. I also invited Xu and Zhu, who were more experienced in qualitative methods, to review and discuss the data with me. When disagreements arose, we returned to the original recordings to verify the participants' expressions. One challenge I faced as a beginner was knowing when data saturation had been reached. To address this, I followed the advice in the methodological literature, conducting additional interviews and documenting my reflections to inform my decisions regarding saturation. |
| --- | --- |
| Translation and back-translation | We consulted methodological literature on translation and back-translation to guide our approach. To minimise individual bias, Xu and I translated the transcripts into English independently, before discussing our versions with Zhu to produce a reconciled draft. For the back-translation, we invited Yi Zhou, a native English speaker of Chinese heritage with no medical training, to review the harmonised quotations. Her background enabled her to capture natural language and cultural nuances without being influenced by technical terminology. We considered her suggestions during team discussions, where we evaluated how best to balance semantic accuracy with cultural appropriateness. Having no prior collaboration with the team, Yi Zhou’s role as an external consultant brought an independent perspective that helped us avoid being constrained by our internal consensus. We also acknowledge several limitations. Translation is an inherently negotiated process, and the final English text may not fully convey the cultural or emotional nuances of the original Chinese quotations. As less experienced researchers, we occasionally deferred to the linguistic or methodological expertise of our more experienced colleagues during the reconciliation process. Although Yi Zhou's lack of medical training reduced technical bias, it may have limited her ability to capture clinical subtleties, necessitating further interpretation by the research team. |

Contextual reflexivity

| Data collection | This study was conducted in a tertiary hospital in Shanghai, China, where the work pace is relatively fast and the overall atmosphere is somewhat tense. Regarding my target participants, young and middle-aged patients with coronary heart disease, I consulted with other staff in the cardiology department. They indicated that patients in typical shared wards might withhold or alter their responses out of concern for self-esteem, privacy and social expectations, or to protect themselves. They might also be reluctant to express negative opinions about the healthcare system if they recognised me as their treating nurse. To address this, I conducted interviews in a private single-patient room and clarified to participants that I was a graduate nursing student and not their responsible nurse, in order to provide a safe and confidential environment. During the interviews, I focused on active listening and encouraging participants to share their genuine experiences. I offered understanding and reassurance when they expressed strong emotions. I also adhered to ethical requirements and obtained informed consent to ensure that participants' rights were fully protected. Conducting face-to-face interviews enabled me to observe participants' tone of voice, facial expressions and body language, helping me to interpret subtle verbal and non-verbal cues. |
| --- | --- |
| Data analysis | Because this study was conducted in China, we considered the participants' occupations, levels of education, and social roles during data analysis, as well as the ways in which traditional Chinese culture, social norms, and values might influence their expressions. Some participants used hypothetical or second-person perspectives to express their true feelings about pain or stress. We also considered whether to code and categorise culture-specific factors. To avoid overemphasising social and cultural aspects, we repeatedly compared the original recordings with the participants' expressions. We also consulted relevant literature and integrated notes taken during the interviews with team discussions. This ensured that our understanding of the cultural and social context was comprehensive and objective. In this way, we embedded the data analysis within the research context to ensure that the findings reflected the participants' lived experiences and captured the meaning within their specific cultural and social backgrounds. Both the second subthemes of Theme One and Theme Two involve these factors, which we believe adds cultural depth to the study. |
| Translation and back-translation | During the translation and back-translation process, I paid particular attention to the cultural expressions, regional dialects and idiomatic language used by the participants. When participants used local dialects or unique expressions, I either consulted friends who were familiar with the dialect or contacted the participants directly to clarify their intended meaning before transcribing into standard Mandarin. This approach helped to preserve linguistic details. The back-translation was conducted by Yi Zhou, a native English speaker of Chinese heritage. Her cultural background enabled her to recognise context-specific language use. Although she has no medical training, this allowed her to focus on natural language rather than technical terminology, which was beneficial for the translation process. While interpreting participants’ words, we continuously balanced semantic fidelity with cultural appropriateness. For example, when handling participants’ emotional expressions, we aimed to remain true to the original meaning to convey their authentic experiences. We consistently returned to the audio recordings to verify the accuracy of translations. Despite these precautions, we acknowledge that some dialects, idioms, or culturally specific meanings may not be fully conveyed in English, representing an inherent limitation of the translation process. |

**Appendix 6** Translators’ qualifications

1. Translation stage:

First author: Jianli Guo

Graduate nursing student; passed the College English Test Band 6 (CET-6); able to read and understand English academic texts and translate quotations;

Third author: Mengqi Xu

Nursing professional; passed the IELTS; studied at an English-instructed university; experienced in qualitative research;

Corresponding author: Lingyan Zhu

Nursing professor; passed the IELTS; experienced in qualitative research.

2. Back-translation stage:

Yi Zhou: Native English speaker of Chinese heritage, fluent in both Chinese and English, not involved in study design or data collection.
